# Temperature and pH-Dependent Response of Poly(Acrylic Acid) and Poly(Acrylic Acid-*co*-Methyl Acrylate) in Highly Concentrated Potassium Chloride Aqueous Solutions

**DOI:** 10.3390/polym12020486

**Published:** 2020-02-21

**Authors:** Aleksander Sinek, Maria Kupczak, Anna Mielańczyk, Marcin Lemanowicz, Shin-ichi Yusa, Dorota Neugebauer, Andrzej Gierczycki

**Affiliations:** 1 Department of Chemical Engineering and Process Design, Faculty of Chemistry, Silesian University of Technology, 44-100 Gliwice, Poland; Oleja96@gmail.com (A.S.); Andrzej.Gierczycki@polsl.pl (A.G.); 2 Department of Physical Chemistry and Technology of Polymers, Faculty of Chemistry, Silesian University of Technology, 44-100 Gliwice, Poland; Maria.Kupczak@polsl.pl (M.K.); Dorota.Neugebauer@polsl.pl (D.N.); 3 Department of Applied Chemistry, Graduate School of Engineering, University of Hyogo, Hyogo 671-2280, Japan; yusa@eng.u-hyogo.ac.jp

**Keywords:** poly(acrylic acid), upper critical solution temperature (UCST), phase transition, star polymer, inorganic salt solutions, KCl

## Abstract

In this study, the phase transition phenomena of linear poly(acrylic acid) (PAA) and linear or star-shaped poly(acrylic acid-*co*-methyl acrylate) (P(AA-*co*-MA)) in highly concentrated KCl solutions were investigated. The effects of polymer molecular weight, topology, and composition on their phase transition behavior in solution were investigated. The cloud point temperature (*T*_CP_) of polymers drastically increased as the KCl concentration (*C*_KCl_) and solution pH increased. *C*_KCl_ strongly influenced the temperature range at which the phase transition of PAA occurred: *C*_KCl_ of 1.0–2.2 M allowed the phase transition to occur between 30 and 75 °C. Unfortunately, at *C*_KCl_ above 2.6 M, the *T*_CP_ of PAA was too high to theoretically trigger the crystallization of KCl. The addition of hydrophobic methyl acrylate moieties decreased the *T*_CP_ into a temperature region where KCl crystallization could occur. Additionally, the hydrodynamic diameters (*D*_h_) and zeta potentials of commercial PAA samples were examined at room temperature and at their *T*_CP_ using dynamic light scattering. The salt concentration (from 1 to 3 M) did not impact the hydrodynamic diameter of the molecules. *D*_h_ values were 1500 and 15 nm at room temperature and at *T*_CP_, respectively.

## 1. Introduction

Stimuli-responsive polymers considerably change their properties, such as their hydrophilic/hydrophobic state and conformation due to small changes in environmental stimuli [1,2] in either a continuous or stepwise manner [3,4,5]. Different system properties may serve as stimuli, such as temperature, pH, redox systems, light, ionic strength, or host–guest interactions. Therefore, stimuli-responsive polymers have been used as drug release agents, flocculants or flotation agents, switchable surfaces, or in chromatography, gene therapy, tissue engineering, biosensors, bioimaging, and optical switching [6,7,8,9,10,11,12,13,14,15,16,17,18]. Only a few publications have been dedicated to polymers with an upper critical solution temperature (UCST), in which the phase transition occurs between 273 and 373 K. In contrast, many studies have been performed on their counterparts with a lower critical solution temperature (LCST), especially in the temperatures close to 309 K since they are meant as drug delivery systems in human body. Moreover, the specific mechanisms involved in phase transitions often restrict certain practical applications of polymers due to their strict requirements, such as salt-free solutions [10,19]. Interestingly, recent publications have reported polymers that exhibit both a UCST and LCST depending on their concentration, such as poly(trimethylene ether)glycol [20,21].

One of the key factors influencing the phase transition of ionic polymers is the presence of dissolved salts, which can be systemized according to the Hofmeister series [22,23]. These series are represented by anions: ${\mathrm{CO}}_{3}^{2-}$ > ${\mathrm{SO}}_{4}^{2-}$ > $S_{2}O_{3}^{2-}$ > $H_{2}{\mathrm{PO}}_{4}^{-}$ > $F^{-}$ > ${\mathrm{Cl}}^{-}$ > ${\mathrm{Br}}^{-}\;$~ ${\mathrm{NO}}_{3}^{-}\;$> $I^{-}$ > ${\mathrm{ClO}}_{4}^{-}$ > ${\mathrm{SCN}}^{-}$ as well as cations: ${\mathrm{Mg}}^{2+}$ > ${\mathrm{Li}}^{+}$ > ${\mathrm{Na}}^{+}$ = $K^{+}$ > ${\mathrm{NH}}_{4}^{+}$ [24]. Generally, the ions to the left of ${\mathrm{Cl}}^{-}$ and $K^{+}$ in the above-mentioned series are called kosmotropes, whereas the ions to the right of ${\mathrm{Cl}}^{-}$ and $K^{+}$ are called chaotropes. These terms originally indicated the capacity of the ions to create or destroy water interactions, respectively [25]. The phase transition mechanism in the presence of these ions involves three main factors: direct interactions between ions and macromolecules, an alteration of surface tension between the macromolecules and the surrounding liquid, and dispersion forces [22]. These dependencies are comprehensively discussed by Kunz and co-workers [24]. The same paper presents other historical attempts to correlate Hofmeister effects with characteristics of ions and solvents, such as the application of salting-out coefficients, Gibbs free energy, entropy of hydration of ions, increasing surface tension, polarizability, partial molar volume, molar refractivities, Jones–Dole viscosity B-coefficients, entropy change of water, or varying the “empty volume of the solvent” [26,27,28,29,30,31].

In this paper, we focus on poly(acrylic acid) (PAA) and its copolymers with methyl acrylate in concentrated KCl solutions. PAA was chosen due to its solubility in water as well as its pH and thermoresponsiveness. KCl was used because the chloride anion defines the boundary between kosmotropes and chaotropes, so its mechanism of impact on macromolecules is not clear. Secondly, KCl has a strong dependence between solubility and temperature and a high exothermic effect on the nucleation process. The aim of this study was to assess the usability of thermoresponsive (co)polymers with a *T*_CP_ in aqueous solution for later use as a trigger for the nucleation of KCl during isohydric crystallization upon cooling. For this purpose, we investigated PAA and its copolymers of methyl acrylate, which differed by molecular weight and topology in order to predict the cloud point temperatures of these polymers. This will allow us to design process conditions to allow a phase transition in the metastable zone of salt solutions at a desired temperature and salt concentration.

## 2. Materials and Methods

### 2.1. Materials

Methyl acrylate (MA, 99%, Sigma-Aldrich, Poznań, Poland), *tert*-butyl acrylate (*t*BuA, 99%, Alfa Aesar, Warsaw, Poland), and anisole (Alfa Aesar, 99%) were dried over molecular sieves and stored in a freezer under nitrogen. Copper(I) bromide (CuBr, 98%, Fluka, Steinheim, Germany) was purified by stirring in glacial acetic acid followed by filtration and washing with ethanol and diethyl ether. Then, solids were dried under vacuum. *N*,*N*,*N*′,*N*″,*N*″-pentamethyldiethylenetriamine (PMDETA, 99%, Sigma-Aldrich, Poznań, Poland), ethyl α-bromoisobutyrate (EBiB, 98%, Sigma-Aldrich, Poznań, Poland), acrylic acid (AA, 99%, Sigma-Aldrich, Poznań, Poland), azobisisobutyronitrile (AIBN, 98%, Sigma-Aldrich, Poznań, Poland), ethanol (EtOH, 98%, Fluka), sodium bicarbonate (NaHCO_3_, anhydrous, Avantor, Gliwice, Poland), and potassium chloride (KCl, VWR Chemicals, Gdańsk, Poland) were used as received without any purification. L1 (poly(acrylic acid), 240,000 Da, 25% w/w water solution) was purchased from Acros Organics. L2 (poly(acrylic acid)*M*_n_ = 25,000 Da) was purchased from Wako Pure Chemicals. Poly(acrylic acid) (PAA) 7100 g/mol and poly(acrylic acid-*co*-methyl acrylate) (P(AA-co-MA)) g/mol was kindly provided by Dr. Shin-ichi Yusa. Pentaerythritol tetrakis(2-bromoisobutyrate) (PTLBr) was obtained according to a previously reported procedure [6]. Samples of commercially available PAA were obtained in the form of a 25% w/w aqueous solution (Sigma Aldrich, analytical grade).

### 2.2. Synthetic Procedures

#### 2.2.1. Synthesis of Linear Poly(Acrylic Acid-*co*-Methyl Acrylate) (P(AA-*co*-MA)) via Free Radical Polymerization

In a test tube, 1.3 g of acrylic acid and 0.17 g of methyl acrylate were dissolved in 1.5 mL of ethanol. Then, 3.91 mg of AIBN was added, and the solution was purged with argon for 30 min to remove oxygen. The reaction mixture was heated to 60 °C and held at this temperature for 17 h. After the reaction, the solution was added dropwise to diethyl ether to precipitate the product and remove unreacted monomers. After the product was dried under vacuum to a constant mass, 0.92 g of the polymer was obtained.

#### 2.2.2. Synthesis of Star-Shaped Poly(Acrylic Acid) (PAA) and P(AA-co-MA) via Atom Transfer Radical Polymerization (ATRP) (Example S1, Scheme 1)

CuBr (24.5 mg; 0.17 mmol), *t*BuA (10 mL; 22.3 mmol), MA (0.04 mL; 0.46 mmol), PMDETA (36 µL; 0.17 mmol), and anisole (3 mL; 30 vol % of monomer) were placed in a Schlenk flask (25mL) and then degassed by three freeze–pump–thaw cycles. Then, pentaerythritol tetrakis(2-bromoisobutyrate) (PTLBr) (41.7 mg; 0.057 mmol/ 4 initiating sites) was added, and the reaction flask was immersed in an oil bath at 70 °C. The reaction was stopped by exposing the reaction mixture to air. Then, it was diluted with CH_2_Cl_2_ and passed through a neutral alumina column to remove the copper catalyst. The mixture was concentrated via rotary evaporation, and the remaining solution was purified by precipitation in cold toluene. The copolymer was isolated by decantation and dried under vacuum at room temperature to a constant mass (yield: 33%). The second step was acidolysis of the obtained copolymer using a previously reported procedure [2].

### 2.3. Instrumentation

Transmittance was measured using a specially designed apparatus comprised of an Agilent Cary 60 spectrometer (MS Spectrum, Warsaw, Poland) and a Quantum Northwest TC-1 temperature controller coupled with specialty software (MS Spectrum, Warsaw, Poland). All samples were freshly prepared prior to measurements. Before each analysis, a quartz cuvette and a Pasteur pipette were preheated in a laboratory dryer at least 10 K above the cloud point temperatures (*T*_CP_) (determined based on trial experiments). The Peltier holder for the cuvette was also preheated. All transmittance measurements were performed with a 1 K/min cooling rate. If the temperature was lower than 283.15 K, the cuvette was continuously washed with dry air to prevent steam condensation on the walls. The hydrodynamic size of macromolecules and their zeta potential were measured using a Malvern Nano-S90 and Malvern Nano-Z apparatuses (Malvern, UK). The measurements were performed at temperatures lower than *T*_CP_ and at the *T*_CP_ determined based on transmittance measurements. In case of atom transfer radical polymerization (ATRP), conversions of monomers were determined by gas chromatography. The chromatograph (6850 Network GC System, Agilent Technologies, Warsaw, Poland) was equipped with a flame ionization detector. Injector and detector temperatures were kept constant at 250 °C (conditions: anisole as internal standard, column initial temperature 40 °C; column final temperature 200 °C). The conversions were calculated by detecting the decrease of the monomer peak area relative to the standard peak area. ^1^H nuclear magnetic resonance (NMR) spectra were obtained using a Bruker DRX-500 500 MHz (Billerica, AM, USA) or Varian Inova 600 MHz spectrometer (Perlan Technologies, Warsaw, Poland) at 26 °C using tetramethylsilane (TMS) as an internal standard with a polymer concentration of 1 wt %, and CDCl_3_ or CD_3_OD (99.8%) as the solvents. The number-average molecular weights (*M*_n_,_SEC_) and dispersity indices (*Đ*) were evaluated by a size-exclusion chromatography (SEC) system composed of an RI-8020 refractive index detector (Tosoh, Tokyo, Japan), SPD-10A UV detector (Shimadzu, Kyoto, Japan), DP-8020 pump (Tosoh, Tokyo, Japan), ERC-3215α degasser (Shodex, Tokyo, Japan), and CTO-10ASVP column oven (Shimadzu, Kyoto, Japan). A α-M column (Tosoh, Tokyo, Japan) was used with a phosphate buffer at pH 9.0 as the eluent and sodium poly(styrene sulfonate) samples as standards. In case of P(*t*BuA-*co*-MA) obtained via ATRP, molecular weights (*M*_n,SEC_, *M*_w,SEC_) and dispersity (*Ð*) indices were determined by size exclusion chromatograph (SEC, 1100 Agilent 1260 Infinity, Perlan Technologies, Warsaw, Poland) equipped with an isocratic pump, autosampler, degasser, thermostatic box for columns, and differential refractometer MDS RI Detector (Perlan Technologies, Warsaw, Poland). Addon Rev. B.01.02 data analysis software (1st edition, Agilent Technologies) was used for data collecting and processing. The SEC calculated molecular weight was based on calibration using linear polystyrene standards (580–300,000 g/mol). A pre-column guard 5 µm 50 mm × 7.5 mm and PLGel 5 µm MIXED-C 300 mm × 7.5 mm (Perlan Technologies, Warsaw, Poland) were used for separation. The measurements were carried out in tetrahydrofuran (THF) (high performance liquid chromatography (HPLC) grade) as the solvent at 40 °C with a flow rate of 0.8 mL/min. Scanning electron microscopy (SEM) analyses were carried out on a VE-9800 (Keyence, Osaka, Japan) with an accelerating voltage of 10 kV. Samples were prepared by dropping a sample solution onto conducting tape, sputtering with Pt, and completely drying at room temperature. The samples were sputtered with Pt using a Quick Coater SC-701MK (Sanyu electron, Tokyo, Japan).

## 3. Results and Discussion

### 3.1. Phase Transition of High Molecular Weight PAA in Aqueous KCl Solutions

**Figure 1:**
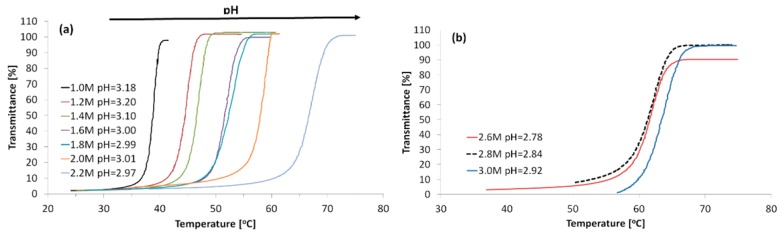
The cloud point temperature (*T*_CP_) curves for 0.25% L1 in KCl solutions with different concentrations without HCl (**a**) and after the addition of 140 μL HCl (**b**).

First, we have studied the influence of KCl concentration (*C*_KCl_) from 1 to 2.4 M (pH between 2.97 and 3.18) and from 2.6 to 3 M (pH from 2.92 to 2.78) on the phase transition of PAA (L1, *M*_w_ = 240,000 g/mol, concentration 0.25% w/w in water). The results revealed that a higher *C*_KCl_ decreased the pH of the obtained solution (Figure 1a). Moreover, as *C*_KCl_ increased, the *T*_CP_ of L1 increased. When *C*_KCl_ was raised to 2.4 M, L1 would no longer dissolve. Surprisingly, the addition of HCl restored the solubility of L1 in highly *C*_KCl_ solutions. Nevertheless, the addition of HCl decreased the pH of the solution, which may have also decreased the *T*_CP_ of the L1 (Figure 1b).

**Figure 2:**
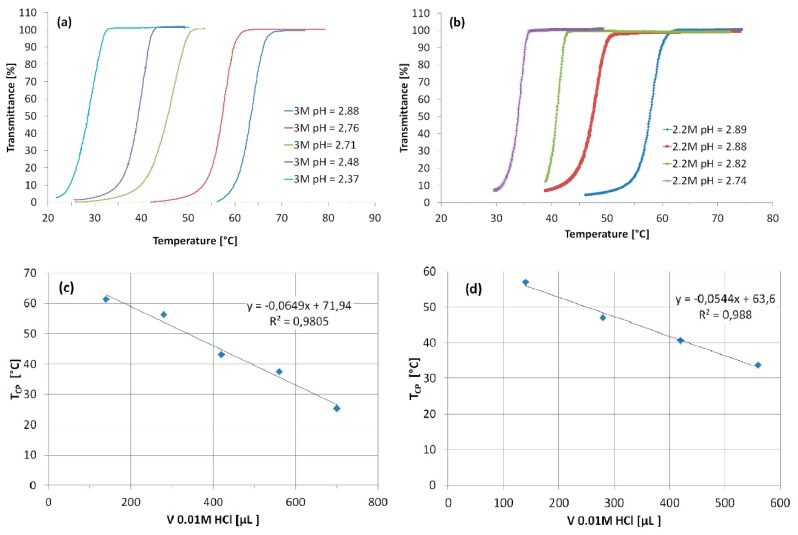
*T*_CP_ curves for 0.25% L1 in a 3 M KCl solution (**a**) and 2.2 M KCl (**b**); the linear decrease of *T*_CP_ of 0.25% L1 as the volume of 0.01 M HCl increased in 3.0 M KCl (**c**) and 2.2 M KCl solutions (**d**).

Further, the behavior of selected 2.2 M and 3.0 M KCl solutions with different pH values showed that the *T*_CP_ of 0.25% L1 decreased linearly with the quantitative addition of HCl (Figure 2).

**Figure 3:**
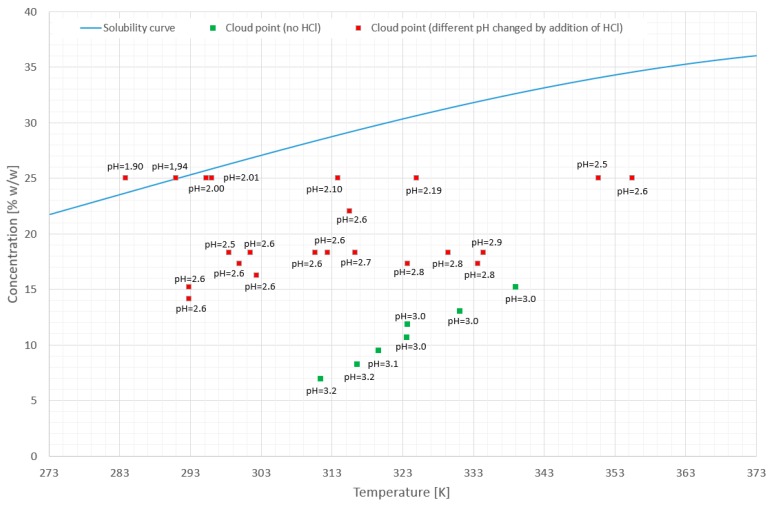
Comparison between the KCl solubility curve and *T*_CP_ of L1 at different pH values.

Figure 3 shows that the presence of HCl and *C*_KCl_ have a crucial influence on the phase transition. A higher *C*_KCl_ broadened the pH range over which this phenomenon occurred. Moreover, this process could be designed to allow it to occur in the metastable zone, which opens up completely new possibilities to control the nucleation process.

### 3.2. Particle Size Distribution of High Molecular Weight PAA

**Figure 4:**
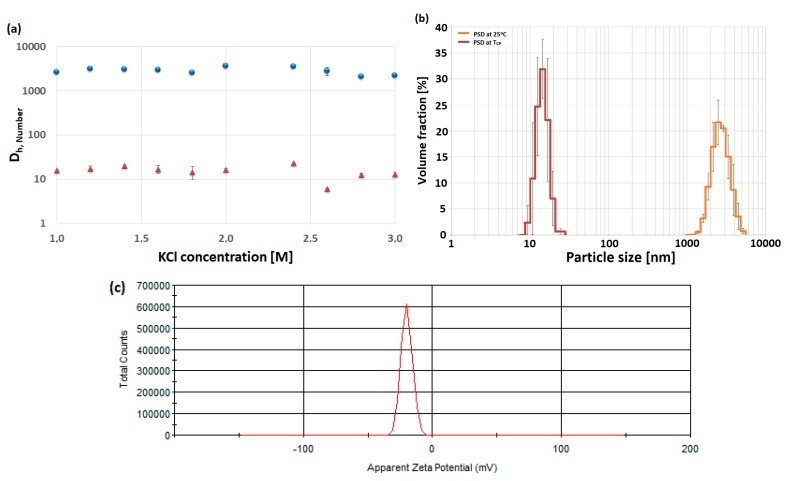
Hydrodynamic diameters (*D*_h_) of 0.25% L1 for various KCl concentrations at 25 °C (dots) and at *T*_CP_ (triangles) (**a**); particle size distribution of 0.25% L1 in 3 M KCl solution at different temperatures (**b**); zeta potential (ZP) of 1% L1 in water at 25 °C (**c**).

As one should expect, the particle sizes of L1 in KCl solutions at 25 °C were larger than above the *T*_CP_ (Figure 4). The hydrodynamic diameters were equal to *D*_h_ = 3557 ± 407 nm and *D*_h_ = 319 ± 4 nm, respectively. This is the result of macromolecules agglomeration due to the strong hydrophobic interactions. The presence of a hydrophobic surface restricts the natural structuring tendency of water, simply by imposing a barrier that prevents the growth of clusters in a given direction. Therefore, water confined in a gap between two such surfaces would not form clusters larger than a certain size. For a narrow gap, this could be a serious limitation and results in the increased free energy of the water in relation to bulk water. Therefore, there is an attraction between hydrophobic surfaces, which is a consequence of water molecules migrating from the gap to bulk water, where there are unrestricted hydrogen-bonding opportunities and lower free energy [32]. In contrast, the macromolecules shrank during their phase transition at the *T*_CP_ from the loose coil into dense globules (just before the agglomeration process occurred). This process can be observed among the others in the work of Zhao and co-workers [19]. Further evidence comes from the analysis of the particle size distribution. At the *T*_CP_, the size distribution is very narrow and ranges between 9 and 30 nm (which represents the suspension of single, dense globules), while at 25 °C, the size distribution is very broad and ranges between 1000 and 6000 nm (which represents the system of multiple agglomerates comprised of macromolecule globules). However, it has to be emphasized that the KCl concentration had no impact on the particle size distribution. This fact proves that the interactions between macromolecules remain unchanged. The average zeta potential (ZP) values were around −1.1 mV for L1 in KCl solutions and −20 mV for L1 in pure water, indicating strong interactions between the carboxyl groups and salt ions.

### 3.3. Scanning Electron Microscopy (SEM) Analysis of High Molecular Weight PAA

**Figure 5:**
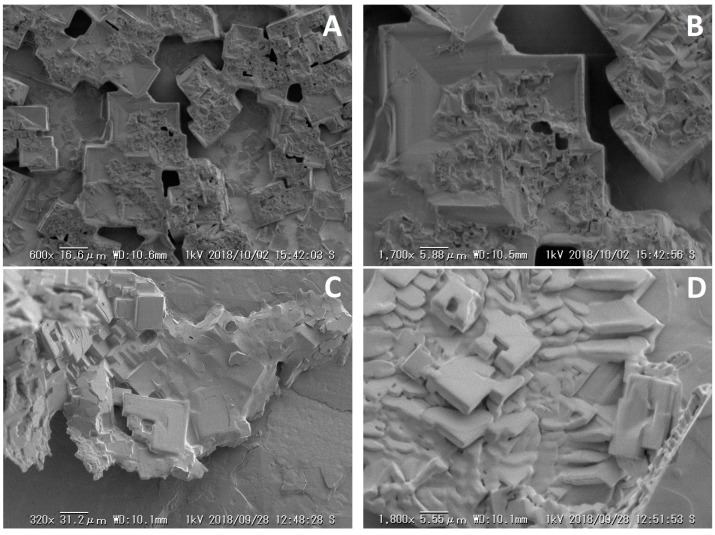
Scanning electron microscopy (SEM) micrographs of pure KCl (**A**,**B**) and L1 in 3 M KCl (**C**,**D**).

The morphology of KCl and KCl with L1 (the starting solution was 0.25% L1 in 3 M KCl) are shown in Figure 5. The comparison of pure KCl and KCl in the presence of PAA showed that the crystal surfaces were covered with a polymeric matrix, which agglomerated them into larger entities. This fact may negatively impact the potential process of crystallization (which could be initialized by the generation of solution imbalance due to the appearance of hydrophobic particles [33]) if the investigated system would be situated below the solubility curve. Usually, the agglomeration of crystals is an undesired process during crystallization: it leads to a lower quality of the product. However, this issue will have to be investigated at significantly larger scales.

### 3.4. Influence of Molecular Weight and Composition of Polymers on T_CP_ in KCl Solutions

**Figure 6:**
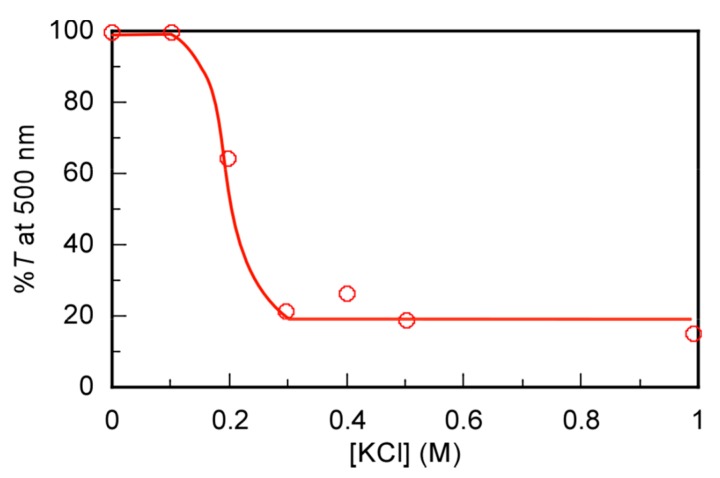
The influence of KCl concentration on the transmittance (%*T*) of the L4 solution (0.25% *w*/*w*).

The influence of the molecular weight of PAA on its *T*_CP_ in 3M KCl was investigated. The first step was to check the solubility of (co)polymers L1–L4 in water and several KCl solutions (from 0.1 to 3 M). In the case of PAA homopolymers, the *T*_CP_ was observed when molecular weight was equal to or higher than 7100 g/mol. The results in Table 1 show that the *T*_CP_ values increased with an increasing molecular weight of PAA. According to the literature, the addition of a hydrophobic unit decreases the *T*_CP_ of LCST-type polymers in aqueous solution and should increase the *T*_CP_ pf UCST-type polymers [34,35]. However, the presence of salt ions in the solution changes the type of interactions between water and macromolecules as well as inter- and intramolecular interactions between macromolecules. Surprisingly, the introduction of hydrophobic methyl acrylate units resulted in a decrease of the *T*_CP_ of a AA/MA copolymer L4, so that its phase transition occurred within the metastable zone of a salt solution (Table 1). Although the dispersity value for this copolymer was quite high (*Đ* = 7.0), we decided to perform the transmittance measurement based on previous results for commercial PAA, which showed no prominent evidence of the impact of broad molecular weight distribution on *T*_CP_. The first transmittance measurement at 0.2 M KCl showed that the *T*_CP_ of the copolymer at pH = 3.1 was 26.2 °C. Moreover, although the L4 copolymer was soluble in water, its solubility decreased as the KCl concentration increased (Figure 6). This indicated that the amount of hydrophobic units was too high, and the composition of copolymer has to be changed. Thus, to gain control over the composition, other copolymers of AA and MA were obtained via ATRP and subsequent post-polymerization modification; however, the molar ratio of AA to MA was increased (*f*_AA_ = 0.98 and *f*_MA_ = 0.02). Additionally, four-arm star-shaped P(AA-*co*-MA) (S1) was obtained in order to investigate if the polymer topology influences the crystallization temperature of KCl.

### 3.5. Synthesis and Characterization of Well-Defined AA/MA Copolymers with Different Topology

**Scheme 1:**
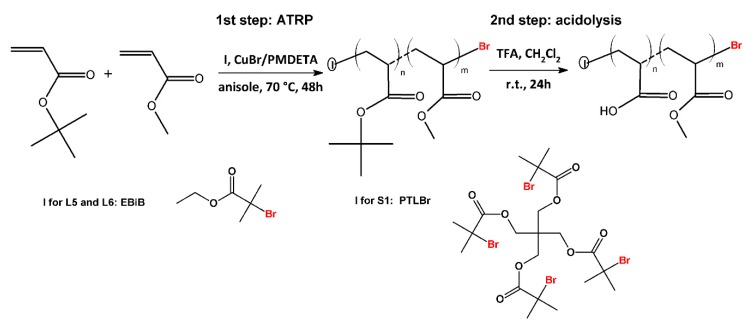
The two-step procedure used to obtain linear and star-shaped acrylic acid (AA)/MA copolymers.

First, *t*BuA/MA copolymers were synthesized by ATRP with low dispersity (*Đ* = 1.18–1.14). Next, post-polymerization modification of the *tert*-butyl groups into acidic groups was conducted (Table 2, Scheme 1), and successful acidolysis was confirmed via ^1^H NMR spectroscopy.

**Figure 7:**
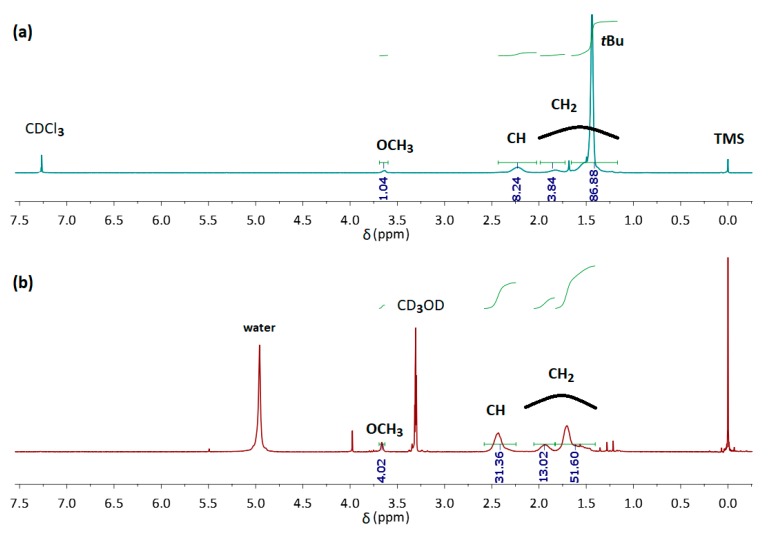
^1^H nuclear magnetic resonance (NMR) (600 MHz) spectra of star-shaped P(*t*BuA-*co*-MA) in CDCl_3_ (**a**) and the product of its acidolysis S1 in CD_3_OD (**b**).

Figure 7 presents a representative ^1^H NMR spectra of star-shaped P(*t*BuA-*co*-MA) and its acidolysis product (S1). The ratios of the methoxy and methine signals’ integral values corresponding to the copolymer before and after acydolysis were constant and confirmed that the cleavage reaction of *tert*-butyl groups was selective and did not affect the ester linkages in MA units. The signal at 1.45 ppm corresponding to the *tert*-butyl protons disappeared after acidolysis, and the presence of the acidic functionality shifted the methine proton resonance signal from 2.23 to 2.43 ppm.

The investigation of T_CP_ for AA/MA copolymers with 2% of MA units content showed that the copolymer L5 was completely soluble in 3M KCl. The measurements were performed for 1% and 0.25% L5 solutions. The pH value for 1% L5 in 3M KCl solution was equal to 1.72, which indicated that a dissociation constant of L5 was high despite the presence of salt. The increase of the molecular weight (copolymer L6) decreased the dissociation of protons, resulting in the appearance of the *T_CP_*.

**Figure 8:**
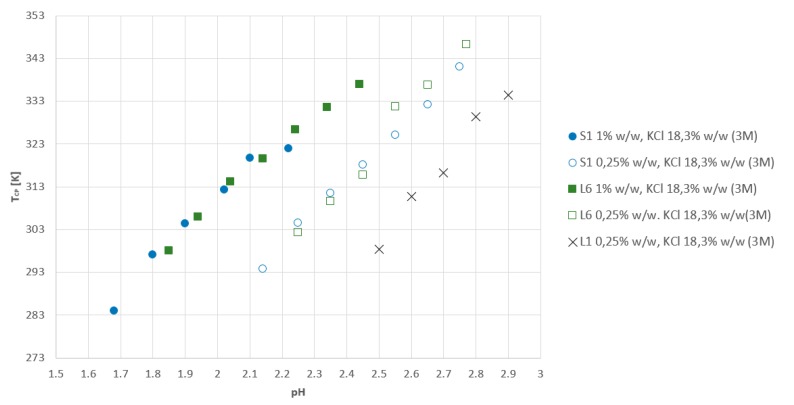
*T*_CP_ values of poly(acrylic acid) (PAA) (L1), linear poly(acrylic acid-*co*-methyl acrylate) (P(AA-*co*-MA)) (L6), and star-shaped P(AA-*co*-MA) (S1) with different concentrations as a function of pH.

The comparison of *T*_CP_ of 3 M KCl solution containing P(AA-*co*-MA) with almost the same molecular weight and composition but different topology showed that the same cloud point temperature could be obtained in the presence of linear (L6) and 4-arm (S1) copolymers but at a different range of pH values depending on the copolymer concentration (Figure 8). A higher copolymer concentration and lower KCl solution pH were applied. Interestingly, the difference in the phase transition temperature triggered by the linear and star-shaped copolymers at the same pH decreased by 20 K with a decrease in copolymer concentration from 1% to 0.25%. Comparing L6 and S1 AA/MA copolymers with nearly the same composition showed that the star-shaped topology decreased *T*_CP_ to 284 K.

## 4. Conclusions

In this paper, the phase transition phenomena of linear poly(acrylic acid) (PAA) and linear or star-shaped poly(acrylic acid-*co*-methyl acrylate) (P(AA-*co*-MA)) in the presence of highly concentrated KCl solutions were investigated. The most important conclusions are:

(1) The *T*_CP_ of PAA strongly depends on pH and salt concentration.
(2) The *T*_CP_ of PAA decreased upon the incorporation of hydrophobic repeating units, such as MA.
(3) A comparison of linear and star-shaped AA/MA copolymers with nearly the same composition showed that the star-shaped topology decreased the *T*_CP_ to 284 K.
(4) It is possible to design process parameters to allow the phase transition to occur in the metastable zone of the salt solution.

The fact that the phase transition phenomenon may occur within the metastable zone implies some very interesting consequences. For example, the solution imbalance introduced by the appearance of hydrophobic particles may trigger the salt nucleation process. This phenomenon could be used as a convenient tool for crystallization process control. Moreover, the presence of macromolecules may influence the solubility of the salt, crystal size distribution, or crystal habit. This research will be continued in order to verify these possibilities.

## Figures and Tables

**Table 1:** Characterization of linear poly(acrylic acid) (PAA) and poly(acrylic acid-*co*-methyl acrylate) (P(AA-*co*-MA)) polymers.

Name	DP_AA_/DP_MA_	M_n,theo_ [g/mol]	M_n,SEC_^a^ [g/mol]	*Đ* ^a^	*T*_CP_^b^	pH
L1 *	-	-	65,600	13.3	56.3	2.8
L2	326/-	25,000	23,500	2.4	49.5	2.8
L3	99/-	7100	7100	1.4	40.5	2.8
L4	96/8	-	45,300	7.0	26.2	3.1

* Commercially available; ^a^ phosphate buffer (pH 9.0) as the eluent and sodium poly(styrene sulfonate) standards ^b^ KCl concentration = 3 M, polymer concentration = 0.25%.

**Table 2:** Characterization of linear and star-shaped P(AA-*co*-MA) samples obtained via atom transfer radical polymerization (ATRP) and subsequent acidolysis.

Name	*t*BuA/MA				AA/MA			
	DP_tBuA_/DP_MA_	M_n,theo_ [g/mol]	M_n,SEC_ [g/mol]	*Đ*	DP_AA_/DP_MA_	M_n,theo_ [g/mol]	M_n,SEC_ [g/mol]	*Đ*
L5	96/2	12,500	13,500	1.18	96/2	7100	n.d.	n.d.
L6	184/3	23,800	38,800	1.16	184/3	13,500	n.d.	n.d.
S1	188/4	24,440	67,100	1.14	188/4	13,900	n.d.	n.d.

Where n.d. indicates that although polymers were soluble in THF and *N*,*N*-dimethylformamide (DMF), the retention times of samples were too high for detection.
